# Neurodevelopmental disorder mutations in the exchange factor DENN/MADD disrupt activation of Rab GTPases

**DOI:** 10.1016/j.jbc.2025.110588

**Published:** 2025-08-12

**Authors:** Maleeha Khan, Rahul Kumar, Jean-François Trempe, Vincent Francis, Emily Banks, Riham Ayoubi, Luis Aguilera Luna, Peter S. McPherson

**Affiliations:** 1 Department of Neurology and Neurosurgery, Montreal Neurological Institute, McGill University, Montréal, Quebec, Canada; 2 Department of Pharmacology & Therapeutics and Centre de Recherche en Biologie Structurale, McGill University, Montréal, Quebec, Canada

**Keywords:** Rab GTPases, neurodevelopmental disorder, DENN/MADD, membrane trafficking, cell biology, guanine nucleotide exchange factor, imaging, protein–protein interaction, genetic disease

## Abstract

DENN/MADD (mitogen-activated protein kinase–activating death domain), a differentially expressed in normal and neoplastic cells (DENN) domain–containing protein functions in membrane trafficking. DENN domain–bearing proteins have guanine nucleotide exchange factor activity toward Rab GTPases. Here, we identify Rab GTPase substrates for DENN/MADD using a cell-based assay involving DENN domain–mediated recruitment of Rab substrates to mitochondria. We confirmed known interactions of DENN/MADD with Rab3A, Rab3B, Rab3C, Rab3D, and Rab27B and identified four new potential substrates, Rab8B, Rab15, Rab26, and Rab37, results confirmed with biochemical experiments. Mutations in the DENN domain of DENN/MADD result in diverse pathophysiological manifestations, ranging from predominant neurological dysfunction to a multisystem disorder. Structural analysis using AlphaFold suggested that these mutations affect DENN/MADD's interaction with Rab GTPases. Introducing such mutations into DENN/MADD's DENN domain influenced the mitochondrial recruitment of Rabs. This study identifies new DENN/MADD protein interactions and cellular pathways, the disruption of which results in human disorders.

Membrane trafficking is crucial for eukaryotic cell function, regulating the distribution and concentration of lipids, proteins, and other macromolecules (1, 2). This process is controlled by Rab GTPases, key players in vesicle formation, motility, and fusion (2, 3, 4). The Rab GTPase family is the largest among the small GTPases, with over 60 members in humans (2). Rab GTPases switch between inactive GDP–bound and active GTP–bound forms (3, 5). GTPase-activating proteins bind to the GTP-bound state, accelerating Rab GTPase activity, thus inactivating the protein. Rabs are removed from the membrane following inactivation, forming a cytosolic complex with guanine dissociation inhibitors (6, 7). Conversely, guanine nucleotide exchange factors (GEFs) bind to the inactive form of Rabs, facilitating the exchange of GDP for GTP. In their active state, Rabs associate with membranes *via* hydrophobic geranylgeranyl groups at their C termini, activating downstream membrane trafficking pathways by recruiting effector proteins that bind specifically to the GTP-bound state of the Rab (8).

Proteins containing a DENN (differentially expressed in normal and neoplastic cells) domain constitute the largest group of GEFs (9). The DENN domain is conserved across evolution, predating the unikont/bikont bifurcation, and there are 18 genes encoding DENN domain proteins in humans (10). DENN/MADD was initially isolated as an open reading frame and named “DENN” (9, 11). It was subsequently shown to interact with the tumor necrosis factor (TNF) receptor and named mitogen-activated protein (MAP) kinase–activating protein containing a death domain (*DENN/MADD*) (12). TNF mediates diverse cellular processes, such as cell survival, proliferation, differentiation, development, and apoptosis *via* MAP kinases (13).

DENN/MADD features a DENN domain at its N terminus, a central serine-rich domain, and a DEATH domain at its C terminus (14). DENN/MADD, also known as RAB3 GEP, exhibits GEF activity toward Rab3A, Rab3C, and Rab3D, regulating calcium-dependent exocytosis of neurotransmitters (15, 16, 17). Furthermore, DENN/MADD functions as a Rab3 effector, aiding the transport of Rab3-containing vesicles to the presynaptic nerve terminal *via* interactions with motor proteins, KIF1Bβ and KIF1A (18). DENN/MADD also has GEF activity toward Rab27, facilitating amylase release in rat parotid acinar (19) and degranulation in natural killer cells (20). DENN/MADD's GEF activity is crucial for Rab27A localization to melanosomes in melanocytes (21, 22). In addition, DEN–MADD activates Rab27A, Rab3B, and Rab3D, facilitating the exocytosis of the hemostatic protein Von Willebrand factor (23). Furthermore, DENN/MADD serves as a GEF for Rab27B in the secretory cascade activated by the calcium-sensing receptor (24). DENN/MADD is known to mediate glucose-induced insulin secretion in pancreatic β-cells; disruption of this pathway can cause type II diabetes (25). In Alzheimer's disease, a reduction in DENN/MADD levels is shown to be associated with neuronal death (26).

Monoallelic DENN/MADD variants have been linked to autism (27) and muscular dystrophy (28), whereas biallelic variants in DENN/MADD result in a neurological disorder or a multisystem disorder (14, 29). Schneeberger *et al.* studied 23 patients with 21 distinct biallelic DENN/MADD variants, finding some overlapping clinical features, such as global developmental delay and/or intellectual disability, along with seizures and hypotonia. The patients were further grouped into two categories based on their phenotypes. Group 1, consisting of 14 patients, presented with a multisystem disorder with severe developmental delay, endocrine and exocrine dysfunction, postnatal failure to thrive, hematological abnormalities, and sensory and autonomic nervous system issues (14). The mutations in this group are spread across the gene and include multiexon deletions, splice site variants, missense mutations, as well as truncated variants (14). On the other hand, group 2, with nine patients, exhibited primarily neurological symptoms, such as mild to severe developmental delay, hypotonia, speech deficits, and seizures (14). Group 2 included missense mutations and truncated variants (14). In conclusion, mutations in DENN/MADD impact a range of cellular functions, and disruptions to its protein levels can lead to a variety of phenotypes.

To fully understand the function of DENN/MADD in Rab-mediated membrane trafficking, we used an unbiased cell-based assay to identify potential Rab-binding substrates for the protein (30). This assay is based on two strategies. First, when GEFs are artificially targeted to specific organelle membranes, including mitochondria, their Rab substrates are relocated to the same membrane, indicating that GEFs primarily govern the localization of Rab GTPases and thus their effectors (31). Second, *in vitro* GEF assays are difficult as it is challenging to purify proteins in their native state. An *in vitro* screen of DENN domain proteins initially assigned a distinct Rab family to the major DENN domain proteins (32). However, later studies identified different or multiple Rab substrates for individual DENN domain–containing proteins (30, 33, 34, 35). These conflicting results can originate from the fact that purifying recombinant Rabs may modify their nucleotide status or lead to their inactivation (30, 36). Furthermore, purified DENN domain proteins may also misfold, leading to a loss of interactions (37).

Therefore, we used a cell-based assay, targeting DENN/MADD to mitochondria to identify potential interacting Rab partners. Our screen found four new potential substrates (Rab8b, Rab15, Rab26, and Rab37) besides the five known substrates (Rab3A/B/C/D and Rab27B). These results were corroborated by coimmunoprecipitation experiments. We then showed how patient mutations in the DENN domain of DENN/MADD belonging to group 1 (catalytic/conserved—P372) and group 2 (unconserved—L346) affect the mitochondrial recruitment of the potential substrates differently. Loss of the catalytic activity because of the P372L mutation abolished the mitochondrial recruitment of all Rabs, whereas the L346P mutation abolished the recruitment for all Rabs except Rab3C and Rab27B, which is further explained through structural biology. In summary, the variety of potential DENN/MADD Rab substrates identified through immunofluorescence, biochemistry, and structural biology helps explain the multisystem disorder observed in DENN/MADD variants.

## Results

### Identification of DENN/MADD Rab GTPase substrates using a cell-based assay

**Figure 1:**
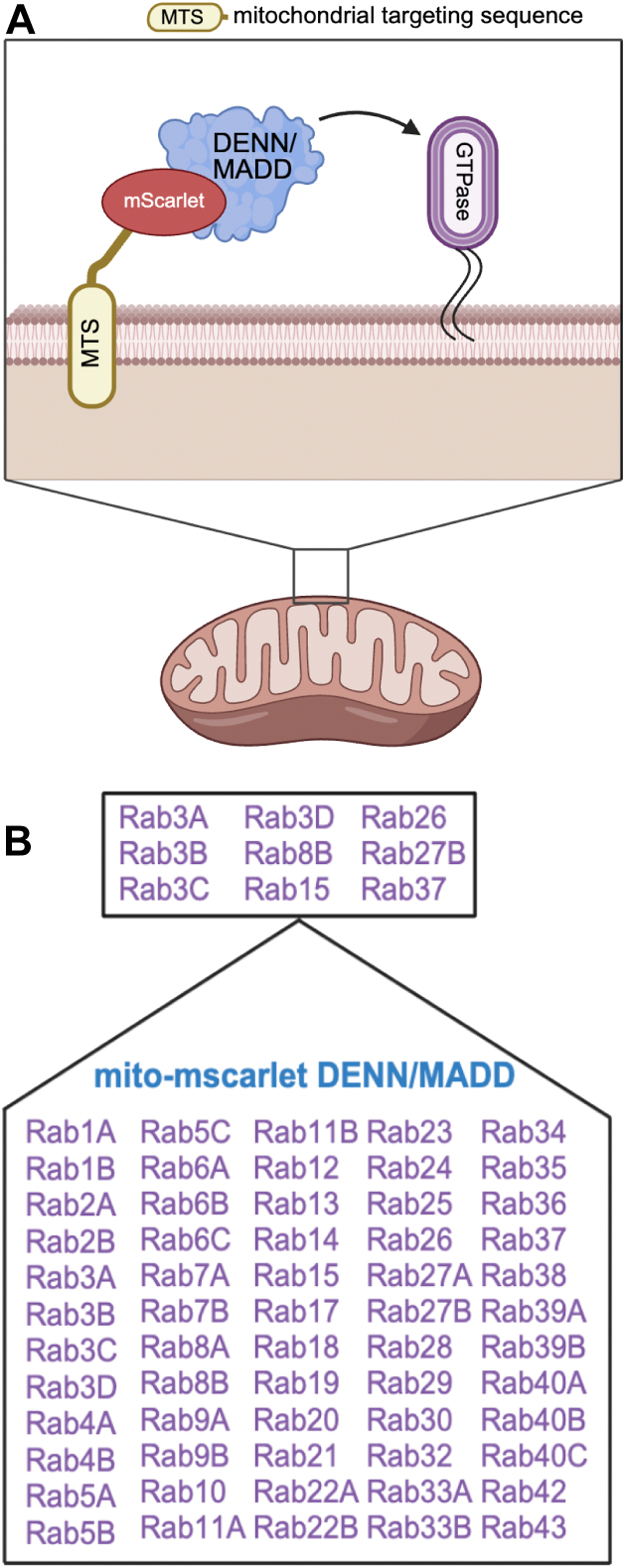
**Cell-based mitochondrial recruitment assay.***A*, schematic model of the cell-based assay. This model is adapted from Rahul *et al.* and generated *via* BioRender. *B*, tabular representation of all the screened and identified Rab GTPases *via* mitochondrial recruitment assay.

**Figure 2:**
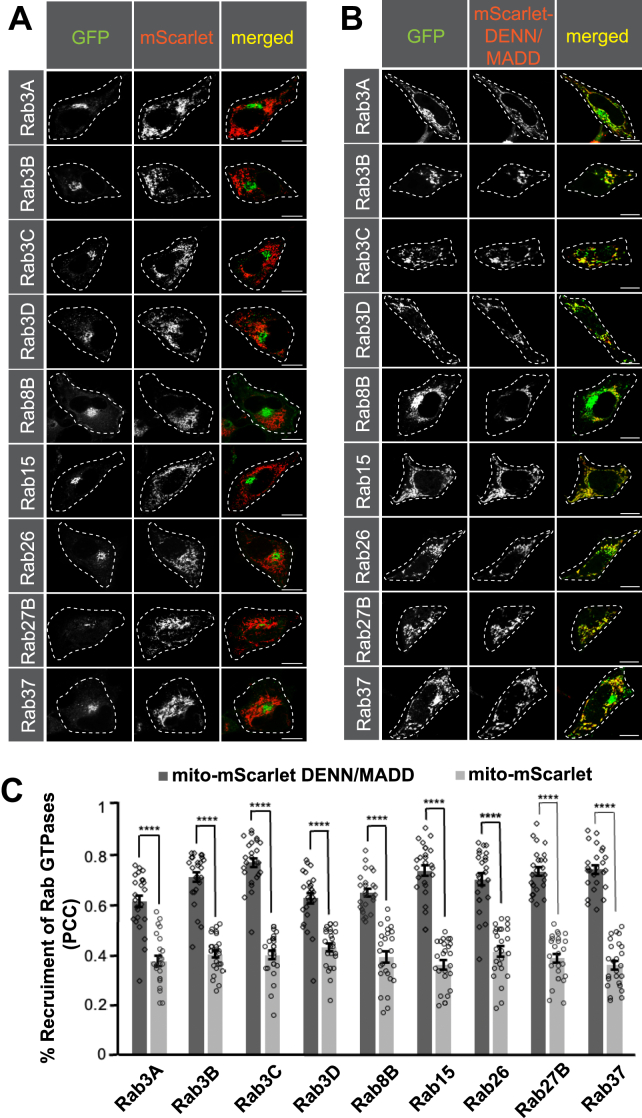
**Mitochondrial-targeted DENN/MADD recruits corresponding Rab GTPases.** HeLa cells cotransfected with GFP-Rabs and mito-mScarlet (*A*) or mito-mScarlet-DENN/MADD (*B*). Scale bars represent 10 μm. *C*, quantification of Rab GTPase colocalization with mito-mScarlet and mito-mScarlet-DENN/MADD using Pearson's correlation coefficient measuring cells from three independent experiments (n = 25) per condition; means ± SEM; unpaired *t* test (for Rab3A, Rab3C, Rab26, Rab27B, and Rab37), Welch *t* test (for Rab8B) and Mann–Whitney *U* test (Rab3B, Rab3D and Rab15); ∗∗∗∗*p* < 0.001. DENN, differentially expressed in normal and neoplastic cells domain; MADD, mitogen-activated protein kinase–activating death domain.

To identify Rab GTPases interacting with DENN/MADD, we used a previously established mitochondrial recruitment assay (30, 37, 38). We designed a mito-mScarlet-DENN/MADD construct, which includes the mitochondrial import signal from the yeast mitochondrial outer membrane protein (Tom70p) followed by mScarlet and full-length DENN/MADD. This allows the construct to be embedded in the mitochondrial outer membrane while DENN/MADD remains accessible to cytosolic proteins (Fig. 1*A*). HeLa cells were cotransfected with mito-mScarlet-DENN/MADD or mito-mScarlet and cotransfected with the individual 60 GFP-Rabs (Fig. 1*B*). We then examined for colocalization between GFP and mScarlet signal. Nine GFP-tagged Rabs colocalized with mito-mScarlet-DENN/MADD (Figs. 1*B* and 2*B*) but negligibly with mito-mScarlet alone (Fig. 2*A*). These include Rab3A/B/C/D, Rab8B, Rab15, Rab26, Rab27B, and Rab37. GFP-Rab5A and GFP-Rab35, included as controls, show minimal colocalization with mito-mScarlet-DENN/MADD (Fig. S1*A*). Quantification of the recruitment of GFP-Rab3A/B/C/D, Rab8B, Rab15, Rab26, Rab27B, and Rab37 to mitochondria using Pearson's correlation coefficient (PCC) showed significantly higher colocalization between the Rabs and mito-mScarlet-DENN/MADD than with mito-mScarlet alone (Fig. 2*C*). Values for colocalization between control Rabs (GFP-Rab5A and GFP-Rab35) and mito-mScarlet-DENN/MADD were similar to the observed PCC values of candidate Rabs with just mito-mScarlet, suggesting minimal colocalization (Figs. 2*C* and S1*B*). DENN/MADD is a known GEF for Rab3A/B/C/D and Rab27A/B and an Rab3A effector (15, 16, 17, 18, 19, 20, 21, 22, 23, 24). However, Rab8B, Rab15, Rab26, and Rab37 are newly identified DENN/MADD-binding partners and could be new GTPases substrates. Rab15 is an endocytic GTPase (39), whereas Rab8B, Rab26, and Rab37 belong to the same functional family of secretory Rabs GTPase as Rab3A/B/C/D and Rab27A/B (40). Furthermore, Rab8B was identified in BioID when DENN//MADD was used as a bait (41). Rab15 was also identified in the screen with DENND1A/B/C and DENND2A/B/C DENN domains, indicating a certain promiscuity in its interactions and functions with DENN domain proteins within the cell (30).

### Biochemical characterization of Rab–DENN/MADD interactions

**Figure 3:**
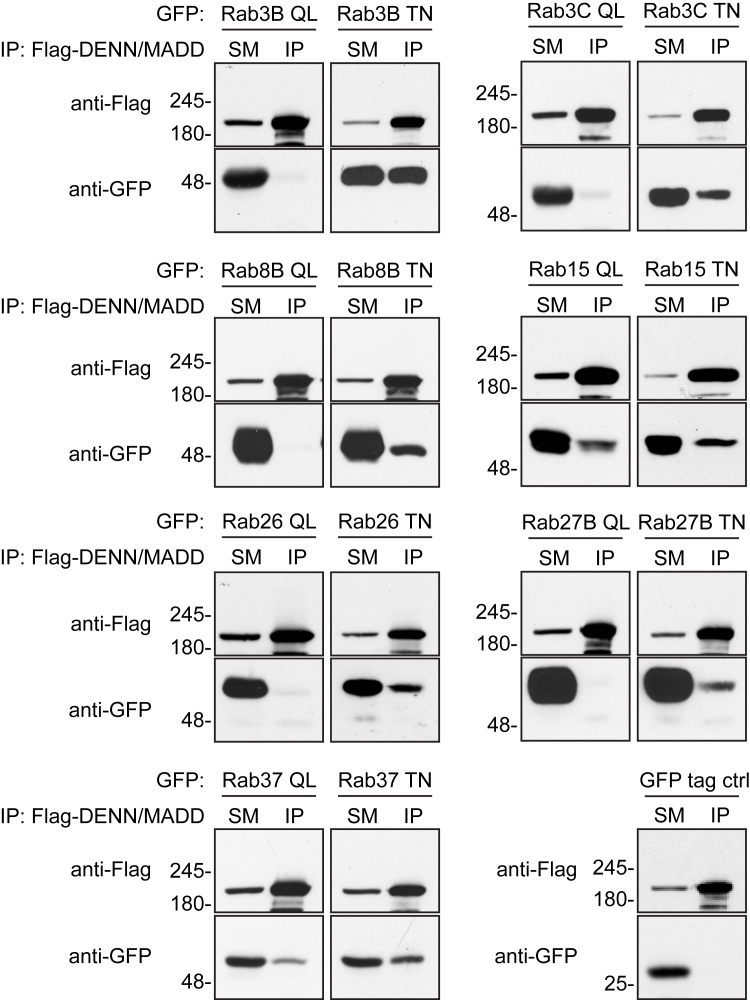
**DENN/MADD preferentially interacts with the inactive (TN) Rab mutants.** Interaction of DENN/MADD with QL and TN mutants of the identified Rabs *via* coimmunoprecipitation. HEK-293T cells were cotransfected with FLAG-DENN/MADD and GFP alone (control/ctrl) or GFP-Rab QL/TN. At 24 h post-transfection, cells were lysed and incubated with FLAG antibody. Bound proteins were identified through immunoblotting using either an anti-GFP antibody to identify active/inactive Rabs or an anti-FLAG antibody that recognizes DENN/MADD. Five percent of the lysate used for coimmunoprecipitation was loaded as starting material (SM). DENN, differentially expressed in normal and neoplastic cells domain; HEK-293, human embryonic kidney 293 cell line; MADD, mitogen-activated protein kinase–activating death domain.

To further characterize the interactions between DENN/MADD and candidate Rab binding partners, we performed coimmunoprecipitation experiments. GEFs bind their substrate GTPases in the GDP-bound inactive form. We thus cotransfected human embryonic kidney 293 (HEK-293) cells with FLAG-DENN/MADD and GFP-Rab QL (constitutively active GTP-bound mutants) or GFP-Rab TN (constitutively inactive GDP-bound mutants) of the candidate Rabs (Fig. 1*B*) as well as controls (Rab5A and Rab35; Fig. S1*B*). The coimmunoprecipitation experiments reveal that DENN/MADD preferentially interacts with the inactive Rab mutants identified in the screen (Rab3BT36N, Rab3CT44N, Rab8BT22N, Rab15T22N, Rab26T81N, Rab27BT23N, and Rab37T43N) compared with the active Rab mutants (Rab3BQ81L, Rab3CQ89L, Rab8BQ67L, Rab15Q67L, Rab26Q127L, Rab27BQ78L, and Rab37Q89L) (Fig. 3), a hallmark of GEFs. Furthermore, there was no interaction between DENN/MADD and the GFP control or control Rab mutants (GFP-Rab5AQL/SN and GFP-Rab35QL/SN) (Figs. 3 and S1*B*). These results support that DENN/MADD is not only a GEF for Rab3 and Rab27 but could also act as a GEF for Rab8B, Rab15, Rab26, and Rab37.

### The patient mutation (DENN/MADD-P372L) in a conserved catalytic residue in the DENN domain disrupts Rab mitochondrial recruitment

A structure of DENND1B in complex with Rab35 revealed seven critical residues responsible for GEF activity (42). Alignment of the DENND1B DENN domain with the DENN domain of DENN/MADD using AlphaFold 2.0 revealed that some of the catalytic residues are conserved in DENN/MADD (Fig. S2). These catalytic residues bind to switch I and II around the nucleotide-binding pocket in Rabs (42). Mutating these residues abolishes the GEF activity of DENN domain proteins toward Rabs (37, 42). Therefore, we selected P372, a conserved/catalytic residue known to interact with the switch II region (42) and generated a missense DENN/MADD mutant resulting in leucine replacing proline, P372L. This residue was also selected because it is mutated (P372L) in biallelic DENN/MADD variants from group 1, as described by Schneeberger *et al.* The two siblings described in this study each carry the P372L missense mutation on one allele, whereas the other allele is truncated and thus missing part of the DEATH domain (14). As previously mentioned, group 1 patients have a multisystem disorder, and two patients containing this mutation show developmental delay, intellectual disability, hypotonia, endocrine and exocrine dysfunction as well as sensory and autonomic dysfunction, failure to thrive and an early death for one (14).

**Figure 4:**
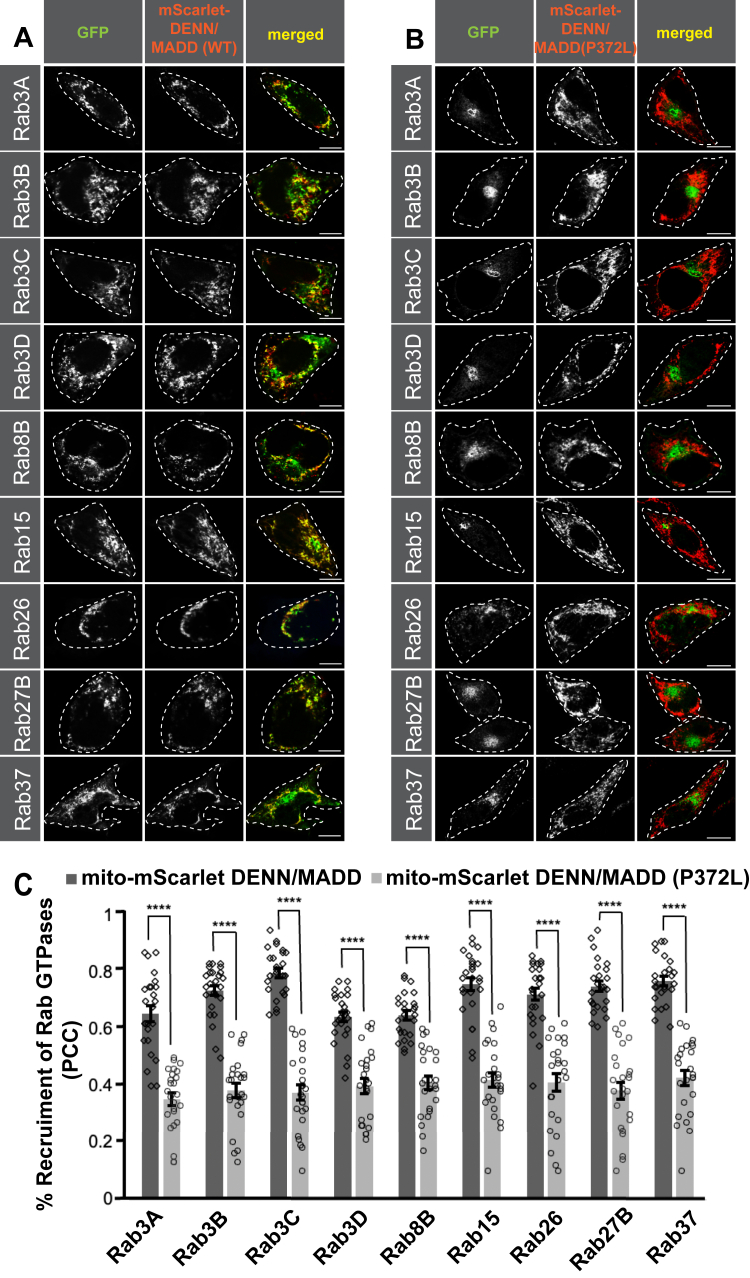
**Cell-based assay reveals disruption of Rab GTPase recruitment because of the mutation in the conserved catalytic residue of DENN/MADD’s DENN domain.** HeLa cells were cotransfected with mito-DENN/MADD (*A*) or mito-scarlet DENN/MADD P372L (*B*) and GFP Rabs. Scale bars represent 10 μm. *C*, quantification of Rab GTPase colocalization with mito-mScarlet-DENN/MADD and mito-scarlet DENN/MADD P372L using Pearson's correlation coefficient measuring cells from three independent experiments (n = 25) per condition; means ± SEM; unpaired *t* test (for Rab3A, Rab8B, and Rab15), Welch's *t* test (for Rab3C, Rab3D, Rab27B, and Rab37) and Mann–Whitney *U* test (for Rab3B and Rab26); ∗∗∗∗*p* < 0.001. DENN, differentially expressed in normal and neoplastic cells domain; MADD, mitogen-activated protein kinase–activating death domain.

We repeated our cell-based assay with the mutation from group 1. We coexpressed mito-mScarlet-DENN/MADD or mito-mScarlet-DENN/MADDP372L along with all nine candidate GFP-Rabs identified in our screen (Fig. 4). We then compared recruitment between mito-mScarlet-DENN/MADD or mito-mScarlet-DENN/MADDP372L and GFP-Rabs (Fig. 4, *A* and *B*). Quantifying the percentage recruitment of GFP-Rabs to mitochondria using PCC identified significantly higher levels of colocalization of GFP-Rabs with mito-mScarlet-DENN/MADD than with mito-mScarlet-DENN/MADDP372L (Fig. 4*C*). Mutating the conserved residue P372 abolished the mitochondrial recruitment of candidate Rabs compared with WT DENN/MADD, illustrating that the GEF activity of DENN/MADD is necessary for the mitochondrial recruitment of potential substrates. This experiment provides the second line of evidence for the potential new DENN/MADD substrates. The candidate Rabs 3A/B/C/D, 8B, 15, 26, 27B, and 37 are expressed in a wide variety of cell types, ranging from neurons (18) to secretary cell (40). Loss of DENN/MADD’s GEF activity leads to potential inactivity of Rab8B, 15, 26, and 37 in addition to Rab3A/B/C/D and Rab27B, which could explain the wide variety of dysfunctional systems seen in group 1 patients.

### The patient mutation (DENN/MADD-L346P) in the DENN domain disrupts Rab mitochondrial recruitment

**Figure 5:**
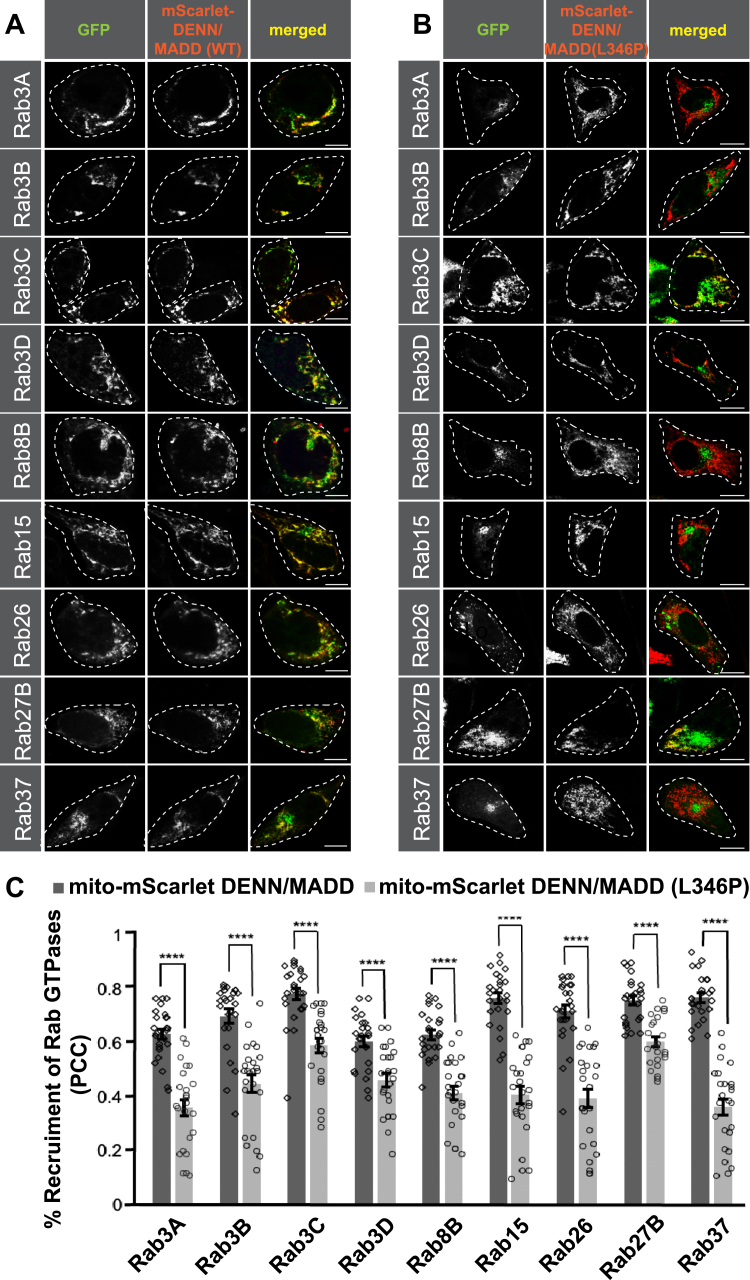
**Cell-based assay reveals disruption of Rab GTPase recruitment because of the mutation in the unconserved residue of DENN/MADD’s DENN domain.** HeLa cells were cotransfected with mito-mScarlet DENN/MADD (*A*) or mito-mScarlet DENN/MADD L346P (*B*) and GFP Rabs. Scale bars represent 10 μm. *C*, quantification of Rab GTPase colocalization with mito-mScarlet-DENN/MADD and mito-mScarlet DENN/MADD L346P using PCC measuring cells from three independent experiments (n = 25) per condition; means ± SEM; unpaired *t* test (for Rab3D, Rab8B, and Rab27B), Welch's *t* test (for Rab3A, Rab15, and Rab37) and Mann–Whitney *U* test (for Rab3B, Rab3C, and Rab26); ∗∗∗∗*p* < 0.001. DENN, differentially expressed in normal and neoplastic cells domain; MADD, mitogen-activated protein kinase–activating death domain; PCC, Pearson's correlation coefficient.

To better understand the phenotype seen in group 2 patients, we repeated our assay with a group 2 patient mutation. We introduced a missense mutation at residue L346, resulting in L346P. This mutation was selected from group 2 (predominantly neurological phenotypes) as it is in the DENN domain but is not a conserved residue involved in GEF catalytic activity (14). This patient contained a biallelic (homozygous) missense mutation of L346P, experiencing developmental delay, intellectual disability, hypotonia, sensory and autonomic dysfunction, and failure to thrive (14). We coexpressed mito-mScarlet-DENN/MADD WT or mito-mScarlet-DENN/MADD L346P with GFP-Rab3A/B/C/D, 8B, 15, 26, 27B, and 37 (Fig. 5, *A* and *B*). There was little to no colocalization between mito-mScarlet-DENN/MADD L346P and GFP-Rabs, except for Rab3C and Rab27. We further quantified the percentage recruitment of GFP-Rabs to mitochondria using PCC and found significantly higher levels of colocalization of GFP-Rabs with mito-mScarlet-DENN/MADD than with mito-mScarlet-DENN/MADD L346P (Fig. 5*C*). Even though the colocalization of Rab3C and Rab27 with mito-mScarlet-DENN/MADD L346P was higher than the other Rabs, it was still significantly lower than the colocalization of Rab3C and Rab27 with mito-mScarlet-DENN/MADD (Fig. 5*C*). To further validate the results of our mitochondrial recruitment assay, we performed coimmunoprecipitation experiments. We cotransfected HEK-293 cells with FLAG-DENN/MADD-L346P and the GFP-tagged QL/TN mutants of Rab3C and Rab27B. We found that DENN/MADD-L346P preferentially interacts with the inactive Rab mutants (Rab3CT44N and Rab27BT23N), similar to WT DENN/MADD, thereby confirming the findings obtained from our cell-based assay (Figs. 5 and S3). In conclusion, this mutation abolished mitochondrial recruitment of all the candidate Rabs except for Rab3C and Rab27B. As previously mentioned, DENN/MADD acts as a GEF for Rab3 playing an essential role in neurotransmitter release (15, 16, 17, 18). The fact that Rab3A, Rab3B, and Rab3D were not recruited might explain why group 2 patients predominantly exhibit a neurological phenotype (14). However, it was interesting that not all candidate Rabs were recruited since 346 is not a catalytic residue, and theoretically, the L346P mutation should not have affected Rab recruitment. To further decipher these results, we performed a structural analysis.

### Structural analysis of DENN/MADD mutants with the candidate Rabs

**Figure 6:**
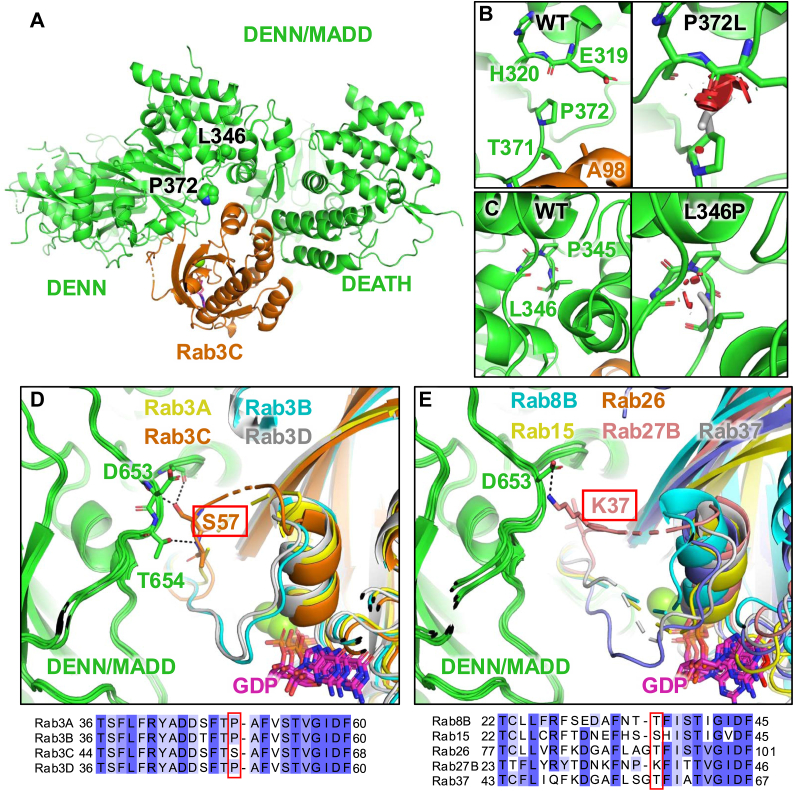
**Structural analysis of DENN/MADD in complex with Rab GTPases.***A*, structural model of DENN/MADD in complex with Rab3C bound to GDP–Mg^2+^ generated by AlphaFold 3. The side chains of Leu346 and Pro372 are shown as *spheres*. *B*, modeling of the P372L mutation. *C*, modeling of the L346P mutation. *D*, superposition of Rab3A, Rab3B, Rab3C, and Rab3D bound to GDP-Mg^2+^ in complex with DENN/MADD. *E*, superposition of Rab8B, Rab15, Rab26, Rab27, and Rab37 bound to GDP–Mg^2+^ in complex with DENN/MADD. DENN, differentially expressed in normal and neoplastic cells domain; MADD, mitogen-activated protein kinase–activating death domain.

To assess the impact of the P372L and L346P mutations on DENN/MADD and its interactions with Rab GTPases, we used AlphaFold 3 to predict the structure of the complexes (43). We modeled full-length human DENN/MADD in complex with each Rab GTPase tested in this study, together with GDP and Mg^2+^. All predictions gave high interface predicted template modeling scores (0.73–0.84) and showed convergence for the five generated models (rmsd = 1.5–2.0 Å), consistent with these proteins making protein–protein interactions. The Rab GTPases all bind the same site on DENN/MADD and interact with both the DENN and DEATH domains (Figs. 6*A* and S4*A*). Pro372 is in close proximity to the switch II helix of the Rab GTPases, and the mutation P372L causes major clashes with the backbone of Glu319 in DENN/MADD as well as changing the conformation of the loop formed by Thr371, which is in contact with Rab GTPases (Fig. 6*B*). This would cause a major conformational change or unfolding of the DENN domain and is consistent with the P372L mutation causing a loss of interaction with all Rab GTPases. On the other hand, the side chain of Leu346 is located in the hydrophobic core of the DENN domain and points toward a helix that is itself interacting with the switch II region of Rab GTPases (Fig. 6*C*). L346P would cause minor clashes with backbone atoms in Ile343 and Pro345 present within the loop harboring Leu346, and modeling of the mutant shows that the protein can adapt to this mutation with minor changes in conformation.

The L346P mutation had a strong effect on the recruitment of most Rabs although the interaction with Rab3C and Rab27 was disrupted to a lesser degree (Fig. 5*C*). We noticed that the switch I region of the Rab, which regulates nucleotide binding (44), generally adopted an “inactive” conformation and interacted with GDP, except in Rab3A, Rab3C, and Rab27B (Fig. 6, *D* and *E*). In those cases, the switch I loop oscillates between two conformations: one where the loop interacts with the DENN domain, thus allowing the nucleotide to be released (consistent with the DENN domain’s role as a guanidine exchange factor), and another conformation closer to GDP (Fig. S4*B*). Furthermore, while the switch I loop in Rab3A is relatively disordered, the same loop in Rab3C and Rab27B forms specific polar contacts with DENN/MADD. In particular, Ser57 in Rab3C makes hydrogen bonds with Asp653 and Thr654 in DENN/MADD (Fig. 6*D*). Ser57 is unique among Rab3 homologs, which otherwise have a Pro residue at this position. Likewise, Lys37 in Rab27B forms a salt bridge with Asp653 in DENN/MADD and is unique among the Rab GTPases tested here (Fig. 6*E*). These additional interactions mediated by the switch I loop in Rab3C and Rab27B would strengthen the interaction with the DENN domain of DENN/MADD. Thus, we hypothesize that the L346P mutation reduces the affinity of DENN/MADD for all Rab GTPases *via* the switch II region, but since Rab3C and Rab27B mediate additional interactions *via* the switch I region, they can sustain an interaction despite the L346P mutation. This is consistent with GEFs generally displaying higher affinity for the nucleotide-free GTPase (45).

## Discussion

Proteins containing a DENN domain form the largest group of GEFs for Rab GTPases (9). The Rab GTPase family, comprising over 60 members in humans, plays a crucial role in membrane trafficking (3). Identifying Rab substrates/interacting partners for DENN domain–bearing proteins is essential for understanding the pathologies caused by disrupted membrane trafficking because of mutations in this family of GEFs (30, 38, 46). Here, we set out to identify the full complement of Rab substrates/binding partners for DENN/MADD, the first identified DENN domain–containing protein (12), using our previously developed cell-based GEF assay to screen for potential Rab substrates (30). DENN/MADD plays a crucial role in TNF-α-dependent signaling pathways as well as membrane trafficking (12, 14). Variants in DENN/MADD result in a multisystem disorder ranging from sensory and autonomic nervous system dysfunction to cognitive and developmental delay as well as endocrine and exocrine disruptions (14). This highlights the diverse roles DENN/MADD plays across various pathways, cell types, and organs. Consequently, the broad range of potential substrates identified in our cell-based assay was not surprising.

We not only confirmed four known DENN/Rab pairs (Rab3A/B/C/D and Rab27B) (16, 23, 24) but also identified potentially novel substrates/partners, including Rab8B, Rab15, Rab26, and Rab37. All the identified Rabs, except for Rab15, belong to a Rab group specialized in secretory vesicle trafficking (40). Rab15 is known as an endocytic GTPase and can act across various endosomal compartments, as it colocalizes with Rab4 and Rab5 on sorting endosomes, as well as with Rab11 on the endocytic recycling compartment (39, 47, 48). It has been demonstrated that DENN/MADD deficiency leads to endocrine dysfunction, specifically defective endocytosis of epidermal growth factor (EGF) (14). Rab5-associated trafficking is required for EGF endocytosis, suggesting a possible indirect effect of Rab15 in EGF endocytosis (49). However, we found that Rab15 acts promiscuity as we previously found this GTPase in screens with DENND1A, B, C and DENND2A, B, C DENN domains (30). The promiscuity of Rab15 might lead to a lack of specificity in its interactions; therefore, this interaction needs to be further studied in detail and confirmed.

DENN/MADD variants have exocrine and neurological dysfunction, and DENN/MADD has been shown to be involved in the release of hormones and neurotransmitters (14, 18, 25). DENN/MADD GEF activity toward Rab27A, Rab3B, and Rab3D is responsible for exocytosis of the hemostatic protein Von Willebrand factor from Weibel–Palade bodies (WPBs) (23). Rab27, Rab3, Rab15, and Rab37 all localize to the WPB membrane, however, only the knockdown of Rab27, Rab3, and Rab15 inhibits WPB exocytosis, demonstrating the commonality among the pathways these Rabs act upon (23, 50). On the other hand, Rab8b was shown to interact with DENN/MADD in a BioID interactome analysis that used proximity biotinylation coupled with mass spectrometry (41). Rab8B is involved in vesicle trafficking in the secretory pathway (51) as well as endocytosis (52). Even though Rab8A and Rab8B share high sequence similarity, only Rab8B, not Rab8A, appeared in the screen, corroborating BioID data and showing the specificity of our screen.

In parotid acinar cells, Rab3D (53), Rab27B (54), and Rab26 (55) localize to secretory granules. DENN/MADD’s GEF activity is essential for Rab27B during granule exocytosis and amylase release from parotid acinar cells (19, 54). Furthermore, Rab26 (56) and Rab3D (57) are also involved in amylase release from acinar cells. Among the secretory Rabs, Rab26 and Rab3D are direct targets of MIST1 (a transcription factor), regulating exocrine granule maturation in acinar cells (58). Rab26 facilitates the formation and maturation of secretory granules, whereas Rab3D anchors these mature granules to the apical cytoskeleton, awaiting the fusion signal to the plasma membrane (58). Biallelic DENN/MADD variants have pancreatic exocrine insufficiency (14, 29), leading to a deficiency of exocrine pancreatic enzymes disrupting digestion (59). Nucleotide polymorphism (SNP) in DENN/MADD is associated with fasting hyperglycemia (60) and type II diabetes because of impaired insulin release (61, 62). DENN/MADD plays a vital role in glucose-induced insulin secretion (25). The release of insulin from pancreatic β-cells, critical for blood glucose regulation, is governed by a series of vesicular trafficking events controlled by Rab GTPases (61). Rab3 and Rab27A produce release-ready secretory granules in pancreatic β-cells, whereas their effectors further regulate insulin secretion (59, 61). The active form of Rab37 is required for insulin secretion *via* autophagy under glucose stimulation (63). Rab37 also facilitates the release of TNF-α from macrophages (64). TNF-α binds to type 1 tumor necrosis factor receptor, activating MAP kinases, a pathway in which DENN/MADD is involved (12).

The potential loss of activity of Rab3A/B/C/D, Rab8B, Rab15, Rab27B, Rab26, and Rab37 may lead to endocrine and exocrine dysfunction. This dysfunction could account for the phenotypes observed in group 1 patients (DENN/MADD-P372L), including growth hormone deficiency, hypothyroidism, and exocrine pancreatic insufficiency. Furthermore, the neurological phenotype seen in this group could be due to the inactivation of Rab3A/B/C/D resulting from a mutated conserved residue in DENN/MADD’s DENN domain, which disrupts GEF activity. On the other hand, group 2 patients predominantly show neurological phenotypes, which could be accounted for due to the loss of Rab3A, Rab3B, and Rab3D activity, evident by the lack of their localization with DENN/MADD-L346P. The reason group 2 does not experience a multisystem disorder, especially endocrine and exocrine dysfunction, could potentially be due to the retained activity of Rab3C and Rab27b, which belong to the two biggest secretory groups of Rabs.

In summary, we identified a new and diverse set of potential Rab substrates/partners for DENN/MADD using an unbiased cell–based assay and biochemistry. We also assessed the impact of patient mutations (L346P and P372L) in DENN/MADD and its interactions with Rab GTPases *via* immunofluorescence and structural biology. Identifying these new potential substrates will help elucidate the complex trafficking mechanisms DENN/MADD is involved in, aiding the understanding and characterization of the wide array of phenotypes across various systems seen in DENN/MADD variants. This will also eventually help provide targets for therapeutic purposes. However, it is crucial to further validate identified Rabs using detailed cell biological studies.

## Experimental procedures

### Cell lines

HeLa and HEK-293T cells were obtained from the American Type Culture Collection.

### Cell culture

Cell lines were grown in Dulbecco's modified Eagle's medium-high glucose (GE Healthcare) containing 10% bovine calf serum (GE Healthcare), 2 mM l-glutamate (Wisent Bioproducts), 100 IU penicillin, and 100 μg/ml streptomycin (Wisent Bioproducts), at 37 °C under 5% CO_2_. The cell lines were routinely tested for mycoplasma contamination using the *mycoplasma* detection kit (Lonza).

### DNA constructs

All 60 GFP-Rab constructs were gifted by M. Fukuda (Tohoku University) (65, 66, 67, 68). QL and TN mutants of Rabs were generated by site-directed mutagenesis (QuickChange Lightning Kit; Agilent) using WT Rab constructs as templates. The primer sequences used for QL and TN mutagenesis are as follows:

Rab3BQ81L (5′-ccggtaccgctctagcccagctgtgtc-3′, 5′-gacacagctgggctagagcggtaccgg-3′),

Rab3BT36N (5′-gaaaaggaaggaattcttcccgacgctgctgttgcc-3′, 5′-ggcaacagcagcgtcgggaagaattccttccttttc-3′), Rab3CQ89L (5′-gtcctgtatctttccaggcctgctgtgtccc-3′, 5′-gggacacagcaggcctggaaagatacaggac-3′), Rab3CT44N (5′-taacggaacaggaaagaatttttgcccacgctgctattgc-3′, 5′-gcaatagcagcgtgggcaaaaattctttcctgttccgtta-3′), Rab8BQ67L (5′-tcggaatctttccaggcccgccgtgtc-3′, 5′-gacacggcgggcctggaaagattccga-3′), Rab8BT22N (5′-ggaacaggaggcagttcttgccaacgccg-3′, 5′-cggcgttggcaagaactgcctcctgttcc-3′), Rab15Q67L (5′-ctggtacctctccagccctgctgtgtc-3′, 5′-gacacagcagggctggagaggtaccag-3′), Rab15T22N (5′-cacagcaggcaattcttgccaaccccggagtcc-3′, 5′-ggactccggggttggcaagaattgcctgctgtg-3′), Rab26Q127L (5′-tcggaaccgctccagaccagctgtgtc-3′, 5′-gacacagctggtctggagcggttccga-3′), Rab26T81N (5′-gcacaagcaggcagttcttccccacaccg-3′, 5′-cggtgtggggaagaactgcctgcttgtgc-3′), Rab37Q89L (5′-gcggaagcgctccagtcctgcagtgtc-3′, 5′-gacactgcaggactggagcgcttccgc-3′), Rab37T43N (5′-ggatcaggaaacagtttttgccgacgcccgag-3′, 5′-ctcgggcgtcggcaaaaactgtttcctgatcc-3′). GFP-Rab5AQ79L and GFP-Rab5AS34N were obtained from J. Presley (McGill). GFP-Rab27B Q78L (catalog no.: 89448) and GFP-Rab27B T23N (catalog no.: 89450) were purchased from Addgene. GFP-Rab35Q67L and GFP-Rab35S22N construction is described in the study by Allaire *et al.*

The following constructs were custom synthesized by SynBio Technologies: mito-mScarlet-DENN/MADD (human DENN/MADD-Variant1, vector:pmScarlet-i_C1), mito-mScarlet (vector:pmScarlet-i_C1), and FLAG-DENN/MADD-V1 (vector-pCMV-Tag2B). Mito-mScarlet-DENN/MADD was then used as a template to create mito-mScarlet-DENN/MADD P372L and mito-mScarlet-DENN/MADD L346P by SynBio Technologies. Whereas FLLAG-DENN/MADD-V1 was used as a template to create FLAG-DENN/MADD-L346P with the following primers:

5′-cgtggcaatgatctacccaccggagtatatgtttcctgtcatcc-3′ and

5′-ggatgacaggaaacatatactccggtgggtagatcattgccacg-3′

### Antibodies

Mouse monoclonal FLAG (M2) (F3165) antibody is obtained from Sigma–Aldrich (Western blot, 1:20,000 dilution). Rabbit polyclonal GFP (A-6455) is obtained from Invitrogen (Western blot, 1:10,000 dilution).

### Cell-based assay

HeLa cells were plated on poly-l-lysine-coated coverslips and cotransfected with individual GFP-Rabs and mito-mScarlet-DENN/MADD (WT, P372L, or L346P) or mito-mScarlet using Lipofectamine Transfection Reagent 2000 (Polyplus). About 24 h post-transfection, cells were fixed with warm 4% paraformaldehyde for 10 min and then washed with PBS three times. Coverslips were mounted onto microscope slides using a fluorescence mounting medium (Dako). Cells were imaged using a Leica SP8 laser scanning confocal microscope with a 63× objective. All images were formatted for publication using Adobe Photoshop (contrast adjusted and a 1-pixel Gaussian blur applied) and subsequently compiled using Adobe Illustrator. To further quantify colocalization between GFP and mScarlet signal, HeLa cells were plated in 96-well plate (PerkinElmer). Cells were cotransfected with individual GFP-Rabs (Rab3AB/C/D, Rab8b, Rab15, Rab26, Rab27B, and Rab37) and mito-mScarlet-DENN/MADD (WT, P372L, or L346P) or mito-mScarlet using Lipofectamine Transfection Reagent 2000 (Polyplus). About 24 h post-transfection, cells were fixed with warm 4% paraformaldehyde for 10 min and then washed with PBS three times. Cells were then imaged with Opera QEHS High-Content Screening System using a 20× water immersion objective obtaining 25 fields of view per well. The quantification of colocalization between GFP and mScarlet signal was performed on all the images using ImageJ colocalization plugin.

### Coimmunoprecipitation

HEK-293T cells were grown to 60% confluency and then transfected with FLAG-DENN/MADD and GFP-tagged constructs using calcium phosphate. After 24 h, the cells were washed with PBS and lysed with a lysis buffer containing 20 mM Hepes, 100 mM NaCl, 0.5 mM dithiothreitol, 10 mM MgCl_2_, 1% Triton X-100, and protease inhibitors (pH 7.4). The lysates were rocked for 20 min at 4 °C and then centrifuged at 305,000*g* for 15 min at 4 °C. The supernatants were incubated with washed protein G-Sepharose beads (GE Healthcare) for 1 h at 4 °C. Samples were then spun down, and 1 mg of the supernatants was incubated with an anti-FLAG antibody and protein G-Sepharose beads for 2 h at 4 °C. The beads were washed three times with the lysis buffer, eluted in Laemmli sample buffer, resolved by SDS-PAGE, and prepared for immunoblotting.

### Immunoblot

Cell lysates were run on large 10% polyacrylamide gels and then transferred onto nitrocellulose membranes. Proteins were visualized using Ponceau staining. Membranes were blocked with 5% milk in Tris-buffered saline with 0.1% Tween-20 (TBST) for 1 h. This was followed by incubation with FLAG and GFP antibodies in TBST with 5% milk for another hour at room temperature. Membranes were then washed with TBST three times for 10 min each and incubated with horseradish peroxidase–conjugated secondary antibody (1:5000 dilution) in TBST with 5% milk for 1 h at room temperature. Following the incubation, membranes were washed with TBST three times, and blots were then developed using Pierce-Enhanced Chemiluminescence Western Blotting substrate (ThermoFisher) onto autoradiographic films.

### Structural analysis

The structure of full-length human DENN/MADD isoform 1 (National Center for Biotechnology Information reference sequence: NP_569826.2, 1588 amino acids) in complex with Rab3a, Rab3b, Rab3c, Rab3d, Rab8b, Rab15, Rab26, Rab27b, and Rab37, as well as GDP and Mg^2+^, were modeled with AlphaFold 3, using a publicly available server (https://alphafoldserver.com/) (43). Each prediction produced five models, which were superposed using PyMOL, version 3.14 (pymol.org). Segments with predicted local distance difference test scores lower than 50 (disordered) were not included in the interpretation. In Figures 6 and S4*A*, only the highest-scoring model for each Rab is displayed. In Fig. S4*B*, all five models are displayed.

### Statistics

All statistical tests were performed using JASP (jasp-stats.org). Normality was checked using Shapiro–Wilk test as well as histograms and QQ plots. *t* Test was used for normally distributed data, whereas the Mann–Whitney *U* test was used for non-normally distributed data. All data are presented as mean ± SEM, with a *p* value less than 0.05 considered statistically significant.

## Data availability

All data are present in the article or the Supporting Information.

## Supporting information

This article contains supporting information.

## Conflict of interest

The authors declare that they have no conflicts of interest with the contents of this article.
