# Supplementary material for: Neurodevelopmental disorder mutations in the exchange factor DENN/MADD disrupt activation of Rab GTPases[image]
Source: J Biol Chem. 2025 Aug 12;301(10):110588. doi: 10.1016/j.jbc.2025.110588 (PMC12495445; doi:10.1016/j.jbc.2025.110588)
Supplement: Supplemental Fig. S1 [file mmc1.pdf]

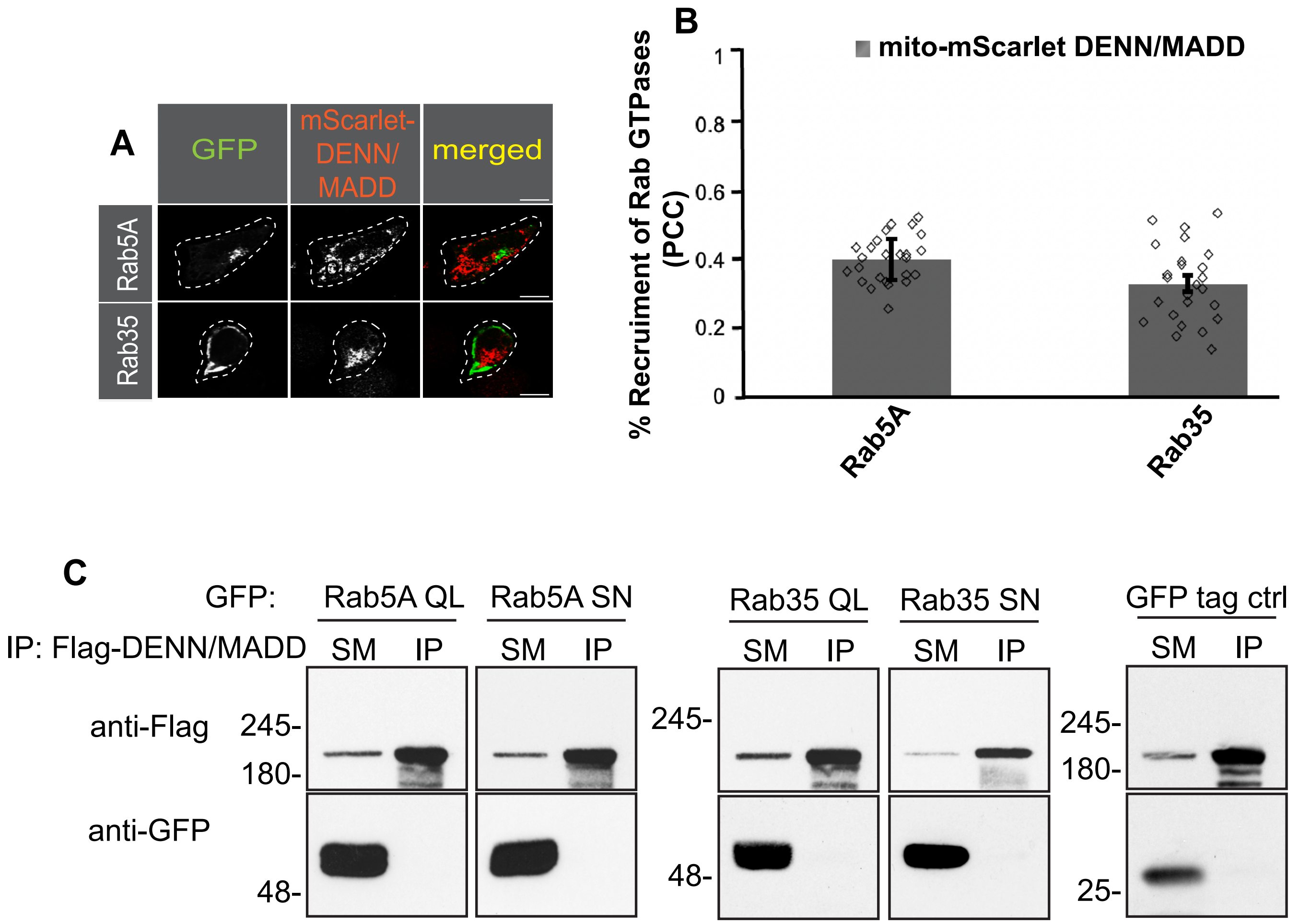

Supplemental Figure S1. Controls: Rab GTPases not recruited via cell-based mitochondrial recruitment assay. (A) HeLa cells co-transfected with GFP-Rabs and mito-mScarlet-DENN/MADD. Scale bars, 10 µm. (B) Quantification of Rab GTPases colocalization with mito-mScarlet-DENN/MADD using PCC (Pearson correlation coefficient) from 3 independent experiments (n=25) per condition; means ± SEM. (C) Interaction of DENN/MADD with QL (active) and SN (inactive) mutants of the Rabs via co-immunoprecipitation. HEK-293T cells were co-transfected with Flag-DENN/MADD and GFP alone (control/ctrl) or GFP-Rab QL/SN. At 24 h post-transfection, cells were lysed and incubated with flag antibody. Bound proteins were identified through immunoblotting using either an anti-GFP antibody to identify active/inactive Rabs or an anti-Flag antibody that recognizes DENN/MADD. 5% of the lysate used for co-immunoprecipitation was loaded as starting material (SM).
