# Supplementary material for: Neurodevelopmental disorder mutations in the exchange factor DENN/MADD disrupt activation of Rab GTPases[image]
Source: J Biol Chem. 2025 Aug 12;301(10):110588. doi: 10.1016/j.jbc.2025.110588 (PMC12495445; doi:10.1016/j.jbc.2025.110588)
Supplement: Supplemental Fig. S2 [file mmc2.pdf]

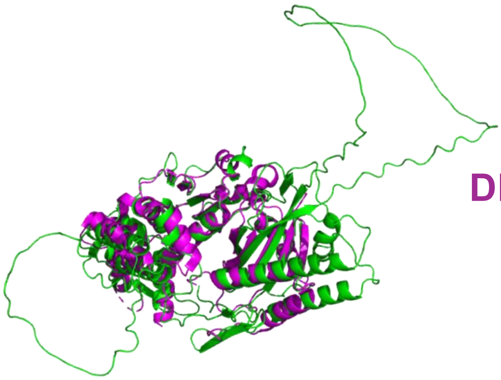

DENN (DENND1B)
DENN (DENN/MADD)

DENND1B residues; DENN/MADD equivalent

I224 = P351
I226 = I353
L233 = A363
Y236 = L366
M241= T371
P242 = P372
Q359 = Q546

Supplemental Figure S2. Structural alignment of DENN domains from DENN/MADD and DENND1B. Structural alignment of DENN/MADD and DENND1B’s DENN domain revealed conserved catalytic residues. The analysis was done using AlphaFold and the image was created with PyMOL.
