# Supplementary material for: Neurodevelopmental disorder mutations in the exchange factor DENN/MADD disrupt activation of Rab GTPases[image]
Source: J Biol Chem. 2025 Aug 12;301(10):110588. doi: 10.1016/j.jbc.2025.110588 (PMC12495445; doi:10.1016/j.jbc.2025.110588)
Supplement: Supplemental Fig. S3 [file mmc3.pdf]

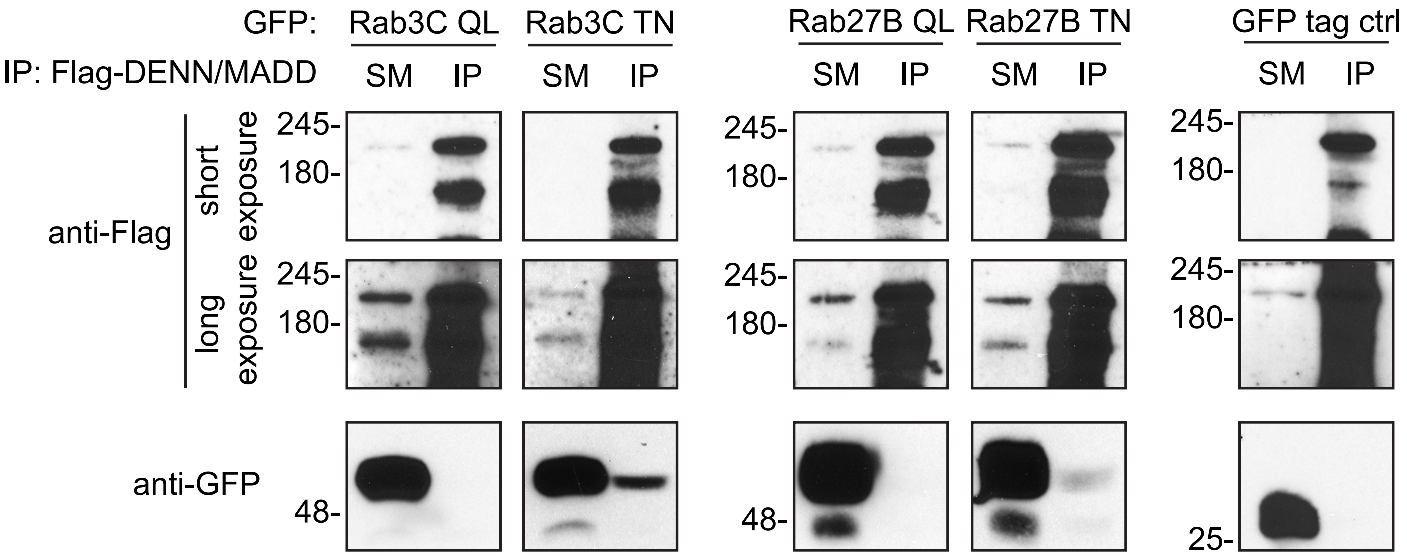

Supplemental Figure S3. DENN/MADD-L346P preferentially interacts with the inactive (TN) Rab mutants. Interaction of DENN/MADD-L346P with QL/TN mutants of the Rab3C or Rab27B via co-immunoprecipitation. HEK-293T cells were co-transfected with Flag-DENN/MADD-L346P and GFP alone (control/ctrl) or GFP-Rab QL/TN. At 24 h post-transfection, cells were lysed and incubated with flag antibody. Bound proteins were identified through immunoblotting using either an anti-GFP antibody to identify active/inactive Rabs or an anti-Flag antibody that recognizes DENN/MADD. 5% of the lysate used for co-immunoprecipitation was loaded as starting material (SM).
