# Supplementary material for: Neurodevelopmental disorder mutations in the exchange factor DENN/MADD disrupt activation of Rab GTPases[image]
Source: J Biol Chem. 2025 Aug 12;301(10):110588. doi: 10.1016/j.jbc.2025.110588 (PMC12495445; doi:10.1016/j.jbc.2025.110588)
Supplement: Supplemental Fig. S4 [file mmc4.pdf]

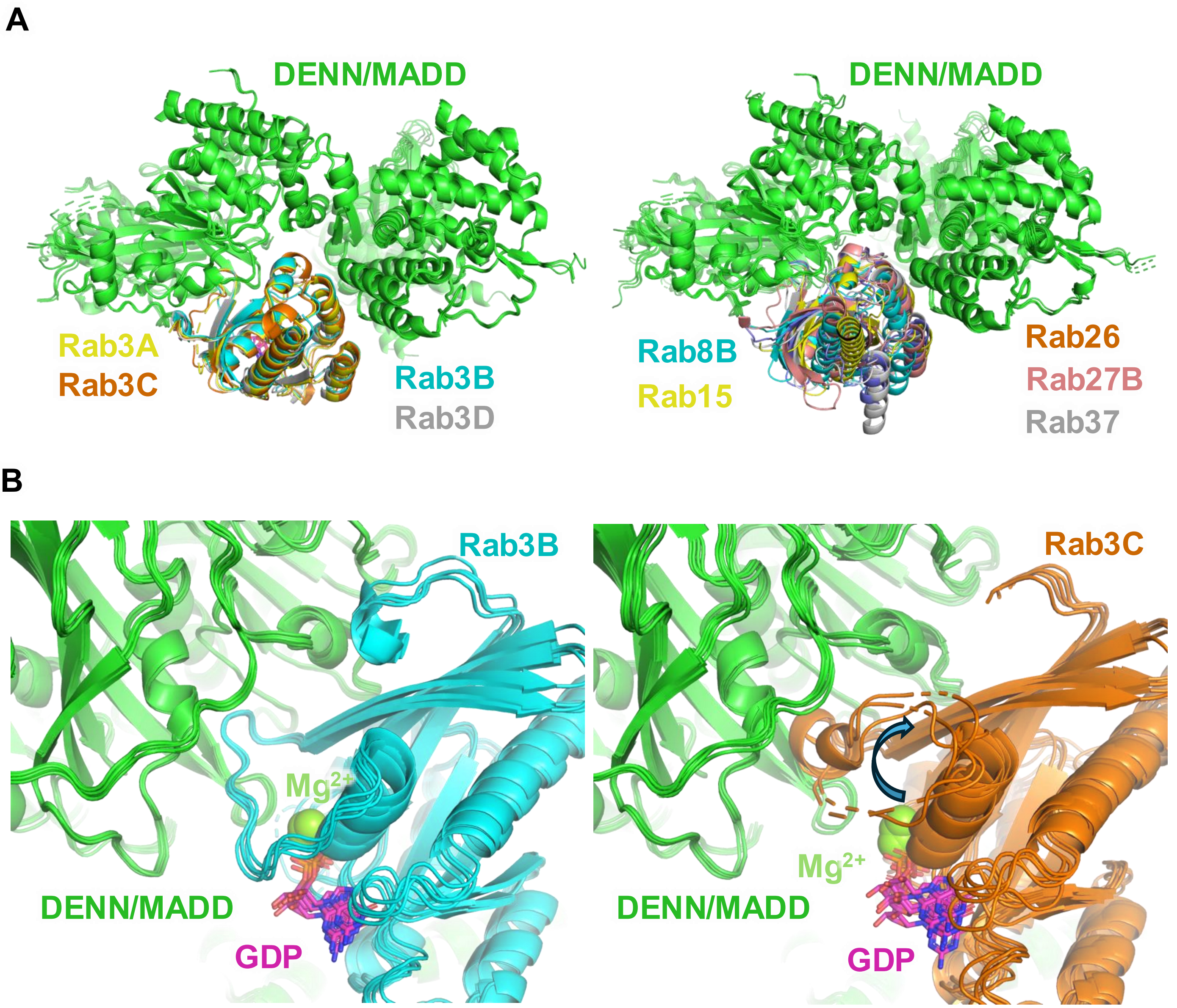

Supplemental Figure S4. Structural analysis of DENN/MADD in complex with Rab GTPases. (A) Structural model of DENN/MADD in complex with different GTPases bound to GDP-Mg^{2+} generated by AlphaFold 3. (B) Structural ensembles of Rab3B (left) and Rab3C (right) bound to GDP-Mg^{2+} and DENN/MADD. The 5 models generated by AlphaFold 3 were superposed.
